# Correlation analysis of long non‐coding RNA TUG1 with disease risk, clinical characteristics, treatment response, and survival profiles of adult Ph^−^ Acute lymphoblastic leukemia

**DOI:** 10.1002/jcla.23583

**Published:** 2021-07-12

**Authors:** Pengyun Zeng, Ye Chai, Chongge You, Lingling Yue, Chongyang Wu, Huiling Chen, Liangliang Li, Jingjing Li, Huan Liu, Yurong Zhang, Tingyong Cao, Yaru Li, Wanli Hu

**Affiliations:** ^1^ Department of Hematology Lanzhou University, Second Hospital Lanzhou China; ^2^ Department of Clinical Laboratory Lanzhou University, Second Hospital Lanzhou China

**Keywords:** long non‐coding RNA TUG1, Philadelphia chromosome‐negative acute lymphoblastic leukemia, reverse transcription‐quantitative polymerase chain reaction, survival, treatment response

## Abstract

**Background:**

Long non‐coding RNA taurine‐upregulated gene 1 (lncRNA TUG1) is reported to be involved in the progression and development of several malignancies; however, its role in Philadelphia chromosome‐negative acute lymphoblastic leukemia (Ph^−^ALL) is unknown. The present study aimed to explore the correlation of lncRNA TUG1 with disease risk, disease condition, and prognosis of adult Ph^−^ALL.

**Methods:**

Total 101 adult Ph^−^ ALL patients and 40 bone marrow (BM) donors were included, followed by detection of BM monocyte cell lncRNA TUG1 expression by reverse transcription‐quantitative polymerase chain reaction. According to the quantiles of lncRNA TUG1 expression in Ph^−^ ALL patients, these patients were divided into four tiers: tier 1 (ranked in 0%~25%), tier 2 (ranked in 25%~50%), tier 3 (ranked in 50%~75%), and tier 4 (ranked in 75%~100%).

**Results:**

LncRNA TUG1 was upregulated in Ph^−^ ALL patients compared with healthy donors. Further analysis indicated that in Ph^−^ ALL patients, higher lncRNA TUG1 tier was correlated with the presence of central nervous system leukemia, increased white blood cell level, and bone marrow blasts. Furthermore, higher lncRNA TUG1 tier was negatively associated with complete remission (CR) within 4 weeks, total CR, and allogeneic hematopoietic stem cell transplant achievement. In addition, higher lncRNA TUG1 tier was associated with decreased disease‐free survival and overall survival, which was further verified to be an independent factor by Cox's regression analysis.

**Conclusion:**

lncRNA TUG1 presents potential to be a novel biomarker for disease risk assessment and survival surveillance in Ph^−^ ALL management.

## Graphical abstract

Long non‐coding RNA taurine‐upregulated gene 1 (lncRNA TUG1) is upregulated and correlates with poor treatment response and unfavrable survival profiles in Philadelphia chromosome‐negative Acute lymphoblastic leukemia (Ph‐ALL) patients, suggesting its potential as a candidate biomarker in ALL managment.

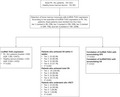

## 1 INTRODUCTION

Acute lymphoblastic leukemia (ALL), as a heterogeneous hematologic disease, is characterized by the abnormal proliferation of immature lymphoid cells in the bone marrow, peripheral blood, and organs.^1^, ^2^ According to the previous study, approximately 60% of ALL cases are diagnosed at younger than 20 years old with the median age of 15 years, and additionally, ALL is considered as the most common form of childhood leukemia, accounting for 75% of pediatric acute leukemias.^3^ The cytogenetics of t(9; 22) chromosomal translocation (also called Philadelphia chromosome (Ph)) is a common chromosomal abnormality in ALL, and there is a wide availability of target agents for Ph^−^positive (Ph^+^) ALL.^3^ As for Ph^−^negative (Ph^−^) ALL, despite recent advancement in the treatment options (such as chemotherapy, hematopoietic cell transplant), part of patients with Ph^−^ ALL still suffer from relapsed/refractory disease and presents poor survival outcomes with the 5‐year overall survival (OS) rates for high‐risk and standard‐risk subgroups of approximately 29% and 54%, respectively.^1^, ^4^, ^5^

Long non‐coding RNA (lncRNA) taurine‐upregulated gene 1 (TUG1), located on chromosome 22q12, binds to polycomb repressive complex 2 and thereby has regulatory effect on the p53‐dependent cell cycle regulatory gene expressions.^6^ Additionally, existing evidences demonstrate that lncRNA TUG1 is regarded as a conserved cancer‐related lncRNA, being aberrantly expressed in multiple tumor tissues, including pancreatic cancer, ovarian cancer, and colorectal cancer.^7^, ^8^, ^9^, ^10^ As for the role of lncRNA TUG1 in hematologic malignancy, lncRNA TUG1 is indicated to be involved in the progression and development of several malignancies, such as multiple myeloma (MM) and chronic lymphocytic leukemia.^11^, ^12^ For example, lncRNA TUG1 is highly expressed in MM patients compared with healthy controls, and clinically, it is correlated with increased MM disease stage, and was of value as a biomarker which helps to facilitate MM diagnosis.^11^ However, the involvement of lncRNA TUG1 in Ph^−^ALL has not been determined yet, therefore, we conducted the present study to explore the correlation of lncRNA TUG1 with disease risk, disease condition, and prognosis of adult Ph^−^ALL.

## 2 METHODS

### 2.1 Subjects

This study retrospectively reviewed 101 adult Ph^−^ALL patients treated in our hospital between January 2014 and December 2019. All analyzed patients met the following criteria: (a) had a diagnosis of primary Ph^−^ ALL by bone marrow morphology, immunology, cytogenetics, and molecular biology (MICM) examinations, (b) aged more than 18 years, (c) had available clinical data and fresh‐frozen pretherapy bone marrow (BM) specimen, (d) had complete treatment remission information in previous 4 weeks and in total, (e) had integrated follow‐up data that were able to used for assessment of disease‐free survival (DFS) and overall survival (OS), and (f) not complicated with other malignancies. Additionally, during the same period, 40 bone marrow donors were included as controls in the current study. This study was approved by Institutional Review Board of our hospital, and written informed consents were collected from all patients (or their families) and healthy donors.

### 2.2 Date collection

Basic clinical data at diagnosis including age, gender, white blood cell (WBC) count, hemoglobin (HGB), blood platelet (PLT), bone marrow blasts, immunophenotype, and central nervous system leukemia (CNSL) were collected from the Computer‐based Patient Record systems (CPRS). Besides, treatment response data including complete remission (CR) within previous 4 weeks and final response status were also extracted from the CPRS. The follow‐up data were collected from the visit records to calculate DFS and OS.

### 2.3 BM sample collection and store

BM samples of Ph^−^ ALL patients were collected before initiation of therapy. After removing of plasma and partial red blood cells, the BM samples were concentrated, followed by adding of dimethyl sulfoxide and TC199 nutrient solution at 4℃; then, the BM samples were put into the polypropylene tube. After that, the tubes were placed in an automatic cooling machine to cool it to −80°C, which were subsequently store in liquid nitrogen until further analysis. The BM samples of health donors were collected when they underwent BM donation, which were treated and stored as described above.

### 2.4 LncRNA TUG1 determination

The BM sample tube was removed from the liquid nitrogen and put into a 40 ℃ water bath for 2 minutes to melt. After that, 10% serum IMDM medium (Gibco) was added into the tube to dilute slowly, and then, Ficoll lymphocyte separation solution (Sigma‐Aldrich) was used to separate BM monocyte cells (BMMCs) for lncRNA TUG1 determination by reverse transcription‐quantitative polymerase chain reaction (RT‐qPCR). In detail, total RNA was extracted from cells using TRIzol™ Reagent (Invitrogen™, Waltham, Massachusetts, USA) and then reversely transcribed using iScript™ cDNA Synthesis Kit (Bio‐Rad, Hercules, California, USA). Following that, qPCR was performed using SYBR^®^ Green Realtime PCR Master Mix (Toyobo) to quantify lncRNA TUG1 expression. In addition, the result was calculated using 2^‐△△Ct^ method with GAPDH as an internal reference. Primers implicated in qPCR were as follows: LncRNA TUG1, forward (5'‐>3'): AGGTAGAACCTCTATGCATTTTGTG, reverse (5'‐>3'): ACTCTTGCTTCACTACTTCATCCAG; GAPDH, forward (5'‐>3'): TGACCACAGTCCATGCCATCAC, reverse (5'‐>3'): GCCTGCTTCACCACCTTCTTGA).

### 2.5 Treatment

All patients received the Chinese Acute Lymphoblastic Leukemia Cooperative Group 2008 (CALLG2008) protocol, which was recommend by Chinese expert panel consensus on diagnosis and treatment of adult acute lymphoblastic leukemia.^13^ The schedules of CALLG2008 comprised of prophase therapy, induction course, consolidation therapy, maintenance therapy, and central nervous system prophylaxis. Details of the CALLG2008 protocol were listed in Supplementary Table S1. During the treatment, if patients were eligible for transplantation, they underwent human leukocyte antigen (HLA)‐matched or haploidentical allogeneic hematopoietic stem cell transplant (allo‐HSCT) after 3 to 5 courses of consolidation therapy; if not, patients continued to receive consolidation and maintenance therapy.

### 2.6 Treatment response evaluation and definition

Morphological analysis of BM cells was performed to evaluate the treatment response on the 28th (±7) day of induction therapy, during which, CR patients were classified as the group with CR within 4 weeks. For patients not achieved CR within 4 weeks, they were given salvage therapy. After induction therapy and salvage therapy, all CR patients were classified as the group with total CR. CR was defined as no circulating blasts or extramedullary disease, trilineage hematopoiesis (TLH) and <5% blasts, absolute neutrophil count (ANC) >1.0 × 10^9^/mL, peripheral blood PLT >100 × 10^9^/mL, and no recurrence for 4 weeks. Relapse was defined as reappearance of blasts in the blood or bone marrow (>5%) or in any extramedullary site after a CR. DFS was defined as the duration from the date of CR to the date of relapse or death in CR status. OS was defined as the duration from the date of diagnosis to the date of death or last follow‐up. Outcome was updated on March 31, 2020.

### 2.7 Statistical analysis

SPSS 24.0 statistical software (IBM) was used for statistical analysis, and GraphPad Prism 8.01 software (GraphPad Software Inc) was used for graphs plotting. Quantitative data were described as mean with standard deviation (SD), or median with interquartile range (IQR). Qualitative data were described as number and percentage (No. (%)). Difference of lncRNA TUG1 expression between Ph^−^ALL patients and health donors was determined by Wilcoxon rank sum test. According to the quantiles of lncRNA TUG1 expression in all patients, patients were divided into four tiers: tier 1 (whose lncRNA TUG1 expression was ranked in 0%~25% among all patients, 0.765 ≤ Tier 1 < 1.990), tier 2 (whose lncRNA TUG1 expression was ranked in 25%~50% among all patients, 1.990 ≤ Tier 2 < 2.801), tier 3 (whose lncRNA TUG1 expression was ranked in 50%~75% among all patients, 2.801 ≤ Tier 3 < 4.261), and tier 4 (whose lncRNA TUG1 expression was ranked in 75%~100% among all patients, 4.261 ≤ Tier 4 < 10.082). Correlation of lncRNA TUG1 tiers with patients’ clinical features, treatment response, and allo‐HSCT was determined by Spearman's rank correlation test or chi‐square test for trend. Kaplan‐Meier curve was used to display DFS and OS, and correlation of lncRNA TUG1 tiers with DFS and OS was determined by log‐rank test. Factors related to DFS or OS were analyzed using univariate and forward stepwise Cox's multivariate proportional hazard regression model. A *P* value less than 0.05 was considered as statistical significance.

## 3 RESULTS

### 3.1 Clinical characteristics of Ph^−^ ALL patients

In Ph^−^ ALL patients, the mean age was 31.7 ± 9.6 years (Table 1). There were 45 (44.6%) females and 56 (55.4%) males included. As for immunophenotype, the number of patients with T‐ALL and patients with B‐ALL were 15 (14.9%) and 86 (85.1%), respectively. Furthermore, there were 95 (94.1%) patients without CNSL and 6 (5.9%) patients with CNSL. The median WBC, HGB, PLT, and bone marrow blasts were 20.7 × 10^9^/L (11.8 × 10^9^/L −36.6 × 10^9^/L), 92.8 g/L (66.4 g/L −108.2 g/L), 46.6 × 10^9^/L (22.3 × 10^9^/L −81.8 × 10^9^/L), and 78.2% (65.3%‐87.1%), respectively. In addition, the number of patients who achieved CR within 4 weeks, patients who achieved total CR, and patients who underwent allo‐HSCT were 73 (72.3%), 91 (90.1%), and 37 (36.6%), respectively. More detailed information of patients’ clinical characteristics was shown in Table 1.

**Table 1:** Patients' characteristics

Items	Ph^−^ ALL patients (N = 101)
Age (years), mean ± SD	31.7 ± 9.6
Gender, No. (%)	
Female	45 (44.6)
Male	56 (55.4)
Immunophenotype, No. (%)	
T‐ALL	15 (14.9)
B‐ALL	86 (85.1)
CNSL, No. (%)	
No	95 (94.1)
Yes	6 (5.9)
WBC (×10^9^/L), median (IQR)	20.7 (11.8‐36.6)
HGB (g/L), median (IQR)	92.8 (66.4‐108.2)
PLT (×10^9^/L), median (IQR)	46.6 (22.3‐81.8)
Bone marrow blasts (%), median (IQR)	78.2 (65.3‐87.1)
CR within 4 weeks, No. (%)	
No	28 (27.7)
Yes	73 (72.3)
Total CR, No. (%)	
No	10 (9.9)
Yes	91 (90.1)
Allo‐HSCT, No. (%)	
No	64 (63.4)
Yes	37 (36.6)

Abbreviations: ALL, acute lymphoblastic leukemia; Allo‐HSCT, allogeneic hematopoietic stem cell transplantation; CNSL, central nervous system leukemia; CR, complete remission; HGB, hemoglobin; IQR, interquartile range; Ph^−^, Philadelphia chromosome negative; PLT, platelet; SD, standard deviation; WBC, white blood cell.

### 3.2 Comparison of lncRNA TUG1 expression between Ph^−^ ALL patients and healthy donors

**Figure 1:**
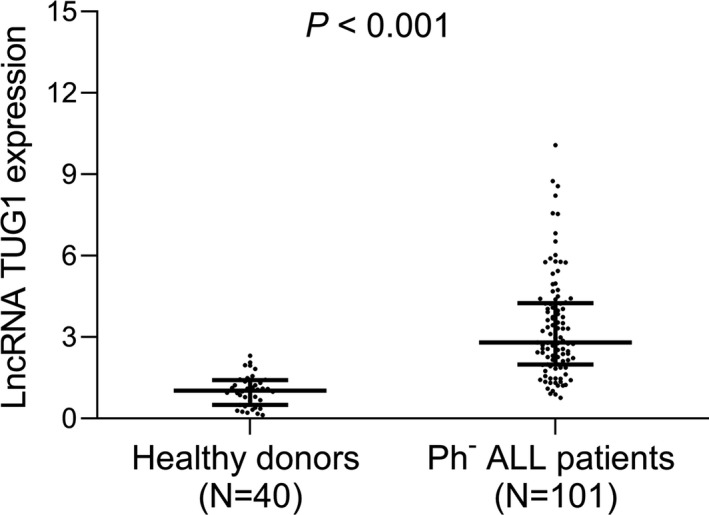
LncRNA TUG1 was upregulated in Ph^−^ ALL patients compared with healthy donors. Comparison of lncRNA TUG1 expression between Ph^−^ ALL patients and healthy donors. LncRNA TUG1, long non‐coding RNA taurine‐upregulated gene 1; Ph^−^ ALL, Philadelphia chromosome‐negative acute lymphoblastic leukemia

LncRNA TUG1 expression was increased in Ph^−^ ALL patients (median: 3.692 (IQR: 1.991‐5.847)) compared with healthy donors (median: 1.033 (IQR: 0.504‐1.415)) (*P* < .001) (Figure 1).

### 3.3 Correlation of lncRNA TUG1 with clinical characteristics in Ph^−^ ALL patients

According to the quantiles of lncRNA TUG1 expression in Ph^−^ ALL patients, patients were divided into four tiers: tier 1 (ranked in 0%~25% among all patients), tier 2 (ranked in 25%~50% among all patients), tier 3 (ranked in 50%~75% among all patients), and tier 4 (ranked in 75%~100% among all patients). Higher lncRNA TUG1 tier was correlated with the presence of CNSL (*P* = .027), increased WBC level (*P* < .001), and bone marrow blasts (*P* = .003), while there was no correlation of lncRNA TUG1 tier with age (*P* = .335), gender (*P* = .615), immunophenotype (*P* = .572), HGB (*P* = .671), or PLT (*P* = .271) (Table 2).

**Table 2:** Correlation of lncRNA TUG1 expression with patients’ characteristics

Items	LncRNA TUG1 expression				*P* value
	Tier 1 (0%‐25%) n = 25	Tier 2 (25%‐50%) n = 25	Tier 3 (50%‐75%) n = 25	Tier 4 (75%‐100%) n = 26	
Age (years), mean ± SD	32.0 ± 6.6	32.0 ± 9.2	29.6 ± 8.3	33.1 ± 13.1	.335
Gender, No. (%)					
Female	11 (44.0)	10 (40.0)	11 (44.0)	13 (50.0)	.615
Male	14 (56.0)	15 (60.0)	14 (56.0)	13 (50.0)	
Immunophenotype, No. (%)					
T‐ALL	3 (12.0)	3 (12.0)	5 (20.0)	4 (15.4)	.572
B‐ALL	22 (88.0)	22 (88.0)	20 (80.0)	22 (84.6)	
CNSL, No. (%)					
No	25 (100.0)	25 (100.0)	22 (88.0)	23 (88.5)	.027
Yes	0 (0.0)	0 (0.0)	3 (12.0)	3 (11.5)	
WBC (×10^9^/L), median (IQR)	14.7 (11.0‐24.1)	12.8 (11.3‐28.1)	26.2 (10.8‐54.2)	33.3 (20.5‐76.1)	<.001
HGB (g/L), median (IQR)	89.4 (67.8‐103.2)	94.7 (73.6‐123.9)	93.3 (70.4‐100.1)	77.7 (63.8‐104.5)	.671
PLT (×10^9^/L), median (IQR)	60.5 (32.0‐86.4)	40.2 (16.8‐80.4)	58.0 (32.4‐87.4)	35.4 (16.1‐70.5)	.271
Bone marrow blasts (%), median (IQR)	72.0 (60.0‐82.2)	78.3 (62.2‐84.7)	77.2 (65.4‐88.5)	83.6 (71.1‐92.7)	.003

Note

Correlation was determined by Spearman's rank correlation test or chi‐square test for trend.

Abbreviations: ALL, acute lymphoblastic leukemia; CNSL, central nervous system leukemia; HGB, hemoglobin; IQR, interquartile range; lncRNA TUG1, long non‐coding RNA taurine‐upregulated gene 1; PLT, platelet; SD, standard deviation; WBC, white blood cell.

### 3.4 Correlation of lncRNA TUG1 with treatment response and allo‐HSCT achievement in Ph^−^ ALL patients

**Figure 2:**
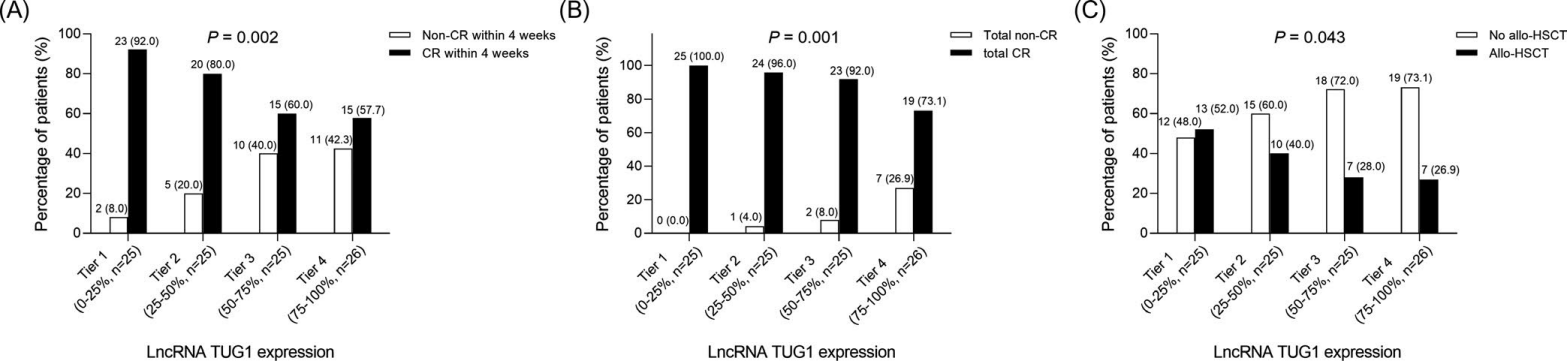
LncRNA TUG1 was negatively correlated with CR and allo‐HSCT. Correlation of lncRNA TUG1 with CR within 4 wk (A), total CR (B), allo‐HSCT (C) in patients with Ph^−^ ALL. LncRNA TUG1, long non‐coding RNA taurine‐upregulated gene 1; Ph^−^ ALL, Philadelphia chromosome‐negative acute lymphoblastic leukemia; CR, complete remission; allo‐HSCT, allogeneic hematopoietic stem cell transplant

There were 23 (92.0%) patients with lncRNA TUG1 Tier 1, 20 (80.0%) patients with lncRNA TUG1 Tier 2, 15 (60.0%) patients with lncRNA TUG1 Tier 3, and 15 (57.7%) patients with lncRNA TUG1 Tier 4 who achieved CR within 4 weeks, suggesting that lncRNA TUG1 tier was negatively associated with CR within 4 weeks (*P* = .002) (Figure 2A). There were 25 (100.0%) patients with lncRNA TUG1 Tier 1, 24 (96.0%) patients with lncRNA TUG1 Tier 2, 23 (92.0%) patients with lncRNA TUG1 Tier 3, and 19 (73.1%) patients with lncRNA TUG1 Tier 4 who achieved total CR, indicating that lncRNA TUG1 tier was negatively associated with total CR (*P* = .001) (Figure 2B). There were 13 (52.0%) patients with lncRNA TUG1 Tier 1, 10 (40.0%) patients with lncRNA TUG1 Tier 2, 7 (28.0%) patients with lncRNA TUG1 Tier 3, and 7 (26.9%) patients with lncRNA TUG1 Tier 4 who underwent allo‐HSCT, suggesting that lncRNA TUG1 tier was negatively associated with allo‐HSCT (*P* = .043) (Figure 2C), which might result from its correlation with CR achievement.

### 3.5 Correlation of lncRNA TUG1 with accumulating survival in Ph^−^ ALL patients

**Figure 3:**
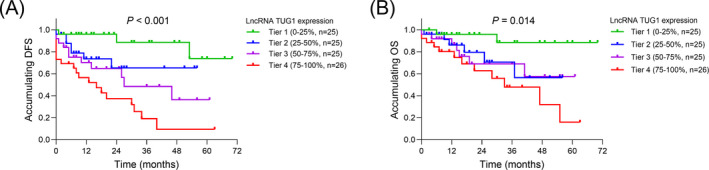
LncRNA TUG1 was negatively associated with survival profiles. Correlation of lncRNA TUG1 with DFS (A) and OS (B) in patients with Ph^−^ ALL. LncRNA TUG1, long non‐coding RNA taurine‐upregulated gene 1; Ph^−^ ALL, Philadelphia chromosome‐negative acute lymphoblastic leukemia; DFS, disease‐free survival; OS, overall survival

We conducted the further analysis to detect the correlation of lncRNA TUG1 with survival profiles in Ph^−^ ALL patients, and found that lncRNA TUG1 tier was negatively associated with accumulating DFS (*P* < .001) (Figure 3A) and accumulating OS (*P* = .014) in Ph^−^ ALL patients (Figure 3B).

### 3.6 Factors associated with DFS in Ph^−^ ALL patients

Univariate Cox's proportional hazard regression analysis indicated that higher lncRNA TUG1 tier (HR = 1.971, *P* < .001), age (≥35 years) (HR = 2.207, *P* = .019), CNSL (HR = 3.764, *P* = .006), and increased WBC at diagnosis (HR = 4.133, *P* < .001) were correlated with decreased DFS, while immunophenotype (B‐ALL vs. T‐ALL) (HR = 0.426, *P* = .020), CR within 4 weeks (HR = 0.165, *P* < .001), and allo‐HSCT (HR = 0.086, *P* < .001) were correlated with increased DFS (Table 3). Further forward stepwise multivariate Cox's regression indicated that higher lncRNA TUG1 tier (HR = 1.470, *P* = .036) and increased WBC at diagnosis (HR = 2.349, *P* = .013) were independent predictive factors for decreased DFS, while CR within 4 weeks (HR = 0.217, *P* < .001) and allo‐HSCT (HR = 0.152, *P* = .003) was independent predictive factors for increased DFS.

**Table 3:** Analysis of factors related to DFS

Items	Cox's proportional hazard regression model			
	*P* value	HR	95%CI	
			Lower	Higher
Univariate Cox's regression				
Higher lncRNA TUG1^a^	<.001	1.971	1.440	2.699
Age (≥35 years)	.019	2.207	1.141	4.271
Male	.438	0.782	0.421	1.455
Immunophenotype (B‐ALL vs. T‐ALL)	.020	0.426	0.207	0.875
CNSL	.006	3.764	1.456	9.736
Increased WBC at diagnosis^b^	<.001	4.133	2.212	7.723
HGB (<100 g/L)	0.058	2.201	0.973	4.979
PLT (<100 × 10^9^/L)	.174	2.266	0.697	7.365
Bone marrow blasts (≥78%)	.100	1.704	0.903	3.213
CR within 4 weeks	<.001	0.165	0.087	0.312
Allo‐HSCT	<.001	0.086	0.026	0.280
Forward stepwise multivariate Cox's regression				
Higher lncRNA TUG1^a^	.036	1.470	1.025	2.108
Increased WBC at diagnosis^b^	.013	2.349	1.195	4.618
CR within 4 wk	<.001	0.217	0.108	0.434
Allo‐HSCT	.003	0.152	0.045	0.521

Abbreviations: ALL, acute lymphoblastic leukemia; Allo‐HSCT, allogeneic hematopoietic stem cell transplantation; CI, confidence interval; CNSL, central nervous system leukemia; CR, complete remission; DFS, disease‐free survival; HGB, hemoglobin; HR, hazard ratio; lncRNA TUG1, long non‐coding RNA taurine‐upregulated gene 1; PLT, platelet; WBC, white blood cell.

^a^ lncRNA TUG1 was categorized as tier 1 (0%‐25%)=1, tier 2 (25%‐50%)=2, tier 3 (50%‐75%)=3, and tier 3 (75%‐100%)=4.

^b^ increased WBC was defined as B‐ALL patients > 30×10^9^/L at diagnosis, and T‐ALL patients > 100×10^9^/L at diagnosis. Factors related to DFS were analyzed by univariate and forward stepwise multivariate Cox's proportional hazard regression model.

### 3.7 Factors associated with OS in Ph^−^ ALL patients

Univariate Cox's regression revealed that higher lncRNA TUG1 tier (HR = 1.755, *P* = .003), age (≥35 years) (HR = 2.233, *P* = .050), increased WBC at diagnosis (HR = 4.925, *P* < .001), and bone marrow blasts (≥78%) (HR = 2.917, *P* = .011) were associated with reduced OS, while immunophenotype (B‐ALL vs. T‐ALL) (HR = 0.340, *P* = .011), CR within 4 weeks (HR = 0.191, *P* < .001), and allo‐HSCT (HR = 0.088, *P* = .001) were correlated with increased OS (Table 4). Forward stepwise multivariate Cox's regression revealed that higher lncRNA TUG1 tier (HR = 3.682, *P* = .002) was an independent predictive factor for reduced OS, while CR within 4 weeks (HR = 0.213, *P* < .001) and allo‐HSCT (HR = 0.169, *P* = .020) were independent predictive factors for increased OS.

**Table 4:** Analysis of factors related to OS

Items	Cox's proportional hazard regression model			
	*P* value	HR	95%CI	
			Lower	Higher
Univariate Cox's regression				
Higher lncRNA TUG1^a^	.003	1.755	1.216	2.533
Age (≥35 y)	.050	2.233	1.001	4.978
Male	.951	0.977	0.456	2.090
Immunophenotype (B‐ALL vs. T‐ALL)	.011	0.340	0.148	0.779
CNSL	.454	1.737	0.409	7.371
Increased WBC at diagnosis^b^	<.001	4.925	2.282	10.629
HGB (<100 g/L)	.337	1.560	0.629	3.867
PLT (<100 × 10^9^/L)	.292	2.171	0.513	9.190
Bone marrow blasts (≥78%)	.011	2.917	1.273	6.684
CR within 4 wk	<.001	0.191	0.089	0.411
Allo‐HSCT	.001	0.088	0.021	0.371
Forward stepwise multivariate Cox's regression				
Higher lncRNA TUG1^a^	.002	3.682	1.628	8.327
CR within 4 wk	<.001	0.213	0.093	0.486
Allo‐HSCT	.020	0.169	0.038	0.754

Abbreviations: ALL, acute lymphoblastic leukemia; Allo‐HSCT, allogeneic hematopoietic stem cell transplantation; CI: confidence interval; CNSL, central nervous system leukemia; CR, complete remission; HGB, hemoglobin; HR, hazard ratio; lncRNA TUG1, long non‐coding RNA taurine‐upregulated gene 1; OS, overall survival; PLT, platelet; WBC, white blood cell.

^a^ lncRNA TUG1 was categorized as tier 1 (0%‐25%)=1, tier 2 (25%‐50%)=2, tier 3 (50%‐75%)=3, and tier 3 (75%‐100%)=4.

^b^ increased WBC was defined as B‐ALL patients > 30×10^9^/L at diagnosis, and T‐ALL patients > 100×10^9^/L at diagnosis. Factors related to OS were analyzed by univariate and forward stepwise multivariate Cox's proportional hazard regression model.

### 3.8 Subgroup analysis: comparison of lncRNA TUG1 expression

LncRNA TUG1 expression was increased in Ph^−^ T‐ALL patients (*P* < .001) (Supplementary Figure S1A) and Ph^−^ B‐ALL patients (*P* < .001) (Supplementary Figure S1B) compared to healthy donors.

### 3.9 Subgroup analysis: correlation of lncRNA TUG1 with treatment response

In Ph^−^ T‐ALL patients, lncRNA TUG1 expression was not associated with CR within 4 weeks (*P* = .181) (Supplementary Figure S2A). Furthermore, in Ph^−^ B‐ALL patients, lncRNA TUG1 expression was negatively associated with CR within 4 weeks (*P* = .014) (Supplementary Figure S2B).

### 3.10 Subgroup analysis: correlation of lncRNA TUG1 with accumulating survival

In Ph^−^ T‐ALL patients, lncRNA TUG1 expression was not correlated with accumulating DFS (*P* = .537) (Supplementary Figure S3A) or accumulating OS (*P* = .800) (Supplementary Figure S3B). As for in Ph^−^ B‐ALL patients, lncRNA TUG1 expression was negatively correlated with accumulating DFS (*P* = .029) (Supplementary Figure S3C), but not accumulating OS (*P* = .066) (Supplementary Figure S3D).

## 4 DISCUSSION

In the present study, we found that (1) LncRNA TUG1 was upregulated in adult Ph^−^ ALL patients compared with healthy donors. (2) In adult Ph^−^ ALL patients, higher lncRNA TUG1 expression was correlated with the presence of CNSL, increased WBC level, and bone marrow blasts. (3) Higher lncRNA TUG1 expression was negatively associated with CR within 4 weeks, total CR, and allo‐HSCT. (4) High lncRNA TUG1 expression was an independent predictive factor for worse DFS and OS.

LncRNAs are a class of non‐coding RNAs; furthermore, recent increasing evidence has indicated that lncRNAs are emerging as key regulators of multiple essential biological processes implicated in human physiological function such as organization of nuclear domains and transcriptional regulation..^14^ However, the biological relevance and pathological of the large majority of lncRNAs remain enigmatic. LncRNA TUG1, as one of lncRNAs, exerts as an imperative role in several human diseases, such as osteoporosis, cardiomyocyte ischemia, and recent researches have demonstrated its implication in the carcinogenesis of several tumors.^7^, ^8^, ^14^, ^15^ For example, lncRNA TUG1 is upregulated in osteosarcoma cells compared with normal osteoblastic cell line, and its knockdown suppresses glucose consumption, lactate production, and cell viability of osteosarcoma cells.^16^ In another clinical study, lncRNA TUG1 is correlated with worse TNM staging in patients with cervical cancer.^17^ As for in hematologic malignancies, lncRNA TUG1 promotes proliferation but inhibits apoptosis in MM via suppressing miR‐29b‐3p.^18^ Nevertheless, the role of lncRNA TUG1 in the pathological progression of Ph^−^ ALL has not been uncovered. Thus, we conducted the present study to investigate the level of lncRNA TUG1 between Ph^−^ ALL patients and healthy donors, and further explore its correlation with disease condition and prognosis in patients with Ph^−^ ALL.

In our present study, we determined the lncRNA TUG1 expression of BM sample from Ph^−^ ALL patients and healthy donors, and found that lncRNA TUG1 was upregulated in Ph^−^ ALL patients compared with healthy donors, suggesting its potential as an indicator for increased ALL risk. Interestingly, in subgroup of Ph^−^ T‐ALL and Ph^−^B‐ALL patients, lncRNA TUG1 expression was also highly expressed in Ph^−^ ALL patients compared with healthy donors. The possible reason might include that according to the previous studies, lncRNA TUG1 overexpression decreased miR‐195 expression, collagen, and aggrecan, leading to the degradation of chondrocyte extracellular matrix and further bone disorder (pain, osteopenia, fracture), and meanwhile, bone disorder associated with bone marrow infiltration is risk factors for ALL, and therefore, lncRNA TUG1 might be implicated in the initiation and progression of ALL.^14^, ^19^ Furthermore, we further detected the correlation of lncRNA TUG1 with clinical characteristics in patients with Ph^−^ ALL, and observed that lncRNA TUG1 was correlated with the presence of CNSL, increased WBC level, and bone marrow blasts, suggesting the correlation of lncRNA TUG1 with poor disease condition in Ph^−^ ALL patients. The possible reasons might include that (a) according to the existing evidence, lncRNA TUG1 was correlated with the level of osteocalcin and osteopontin, increased systematic inflammation level, and deteriorative bone injury, and therefore, lncRNA TUG1 might promote the susceptibility of CNSL, release of WBC, and increase the occurrence of bone marrow blasts in Ph^−^ ALL patients.^14^, ^15^, ^20^, ^21^ (b) Additionally, lncRNA TUG1 might promote cell proliferation, invasion but repress cell apoptosis via targeting aurora kinase A in ALL as in AML, and therefore, lncRNA TUG1 was positively correlated with poor disease condition in Ph^−^ ALL patients, which needed to be further investigated by cellular experiments.^6^

Furthermore, we observed that lncRNA TUG1 was negatively associated with CR within 4 weeks, total CR, and allo‐HSCT in Ph^−^ ALL patients. In addition, we also determined the correlation of lncRNA TUG1 with long‐term prognosis in Ph^−^ ALL patients, and the results exhibited that lncRNA TUG1 independently predicted worse DFS and OS, implying the role of lncRNA TUG1 as a potential biomarker in Ph^−^ ALL management. Interestingly, in subgroup analysis, lncRNA TUG1 was negatively associated with CR within 4 weeks and accumulating DFS only in Ph^−^ B‐ALL patients, which might be due to limited sample size in subgroup. In addition, in the present study, Ph^−^ ALL patients were divided into four tiers according to the lncRNA TUG1 quartile, and we speculated that it might be more convenient to apply lncRNA TUG1 into clinical practice if lncRNA TUG1 expression was divided into high/low expression according to median value. The possible reasons might include that (a) recent several researches had connected the high lncRNA TUG1 expression with increased drug resistance to chemotherapy (such as adriamycin, cisplatin, gemcitabine) in the treatment of several malignancies, and therefore, we speculated that lncRNA TUG1 might enhance drug resistance, leading to poor treatment response and further undesirable long‐term survival profiles in Ph^−^ ALL patients. (b) In addition, according to the prior study, central nervous system relapse was a principal cause of treatment failure in management of Ph^−^ ALL, and according to the prior observation, lncRNA TUG1 was correlated with the presence of CNSL, contributing to poor prognosis in Ph^−^ ALL patients.^22^ (c) Furthermore, based on the previous evidence, allo‐HSCT is considered to be part of post‐remission consolidative therapy, and therefore, for patients with higher lncRNA TUG1 expression, the lower rate of CR was achieved, and the reduced application of allo‐HSCT was conducted.

Our study existed some limitations as follows: (a) Firstly, considering that our study was a retrospective single‐center study with a relatively small sample size, selection bias and relatively low statistical power might exist and further prospective studies with more patients from multiple regions were needed for validation. (b) Secondly, our present study did not include the underlying mechanism of lncRNA TUG1 in the pathology of ALL; therefore, further functional experiments were needed. (c) Thirdly, all patients might receive different treatment applications including prophase therapy, induction course, consolidation therapy, maintenance therapy, and central nervous system prophylaxis according to their clinical presentation (based on CALLG2008 protocol), which might lead to bias in the current study. (d) The present study did not include Ph^+^ ALL patients, and therefore, further studies included these patients were needed for validating the clinical role of lncRNA TUG1. (e) Considering our study was a retrospective study with difference sample size in case group and controls, further prospective studies included the same sample size in case group and control were needed for validation. (f) The patients included were all adult Ph^−^ ALL; however, considering ALL was common among children, and therefore, further studies needed to be conducted for results validation in samples of children.

In conclusion, lncRNA TUG1 is upregulated and correlates with poor disease condition, treatment response, and survival profiles in Ph^−^ ALL patients, implying the potential of lncRNA TUG1 as a useful biomarker in ALL management.

## Supporting information

Fig S1

Fig S2

Fig S3

Table S1

Supplementary Material
