# Supplementary figures and images for: Correlation analysis of long non‐coding RNA TUG1 with disease risk, clinical characteristics, treatment response, and survival profiles of adult Ph− Acute lymphoblastic leukemia
Source: J Clin Lab Anal. 2021 Jul 12;35(8):e23583. doi: 10.1002/jcla.23583 (PMC8373340; doi:10.1002/jcla.23583)

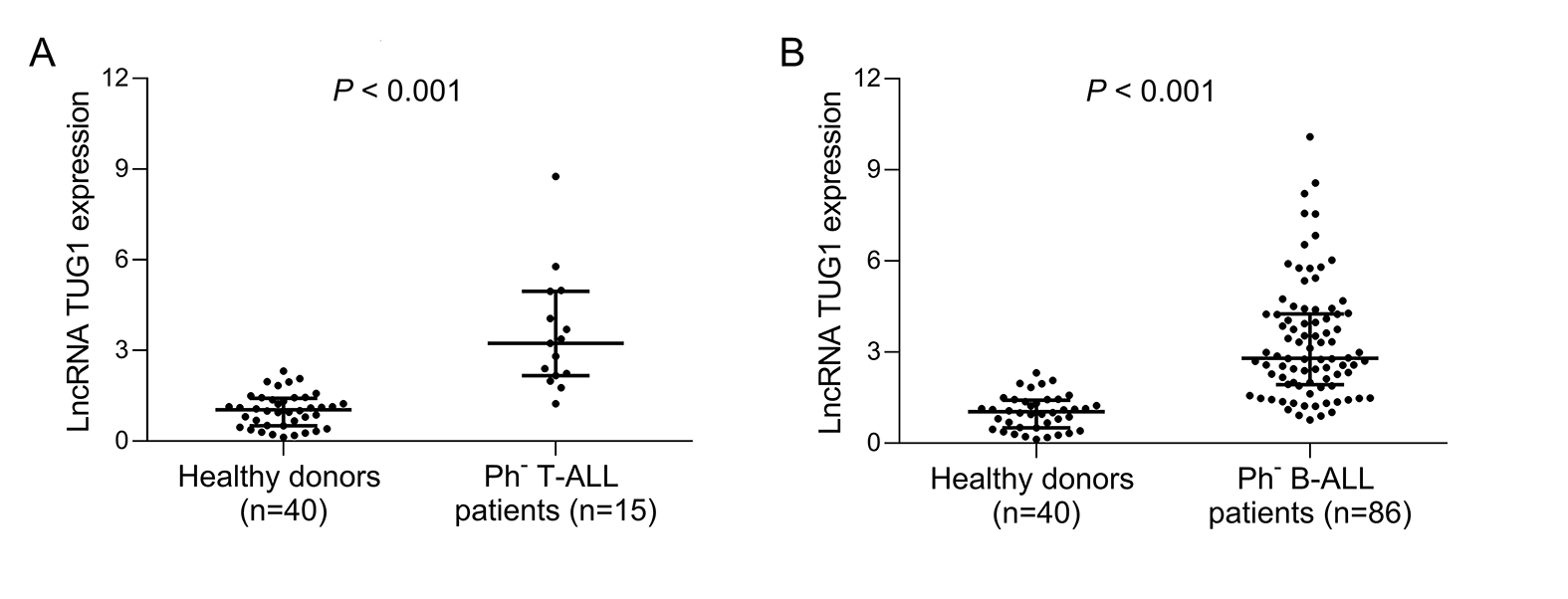

Supplement: Supplementary file 1 — Fig S1 [file JCLA-35-e23583-s004.tif]

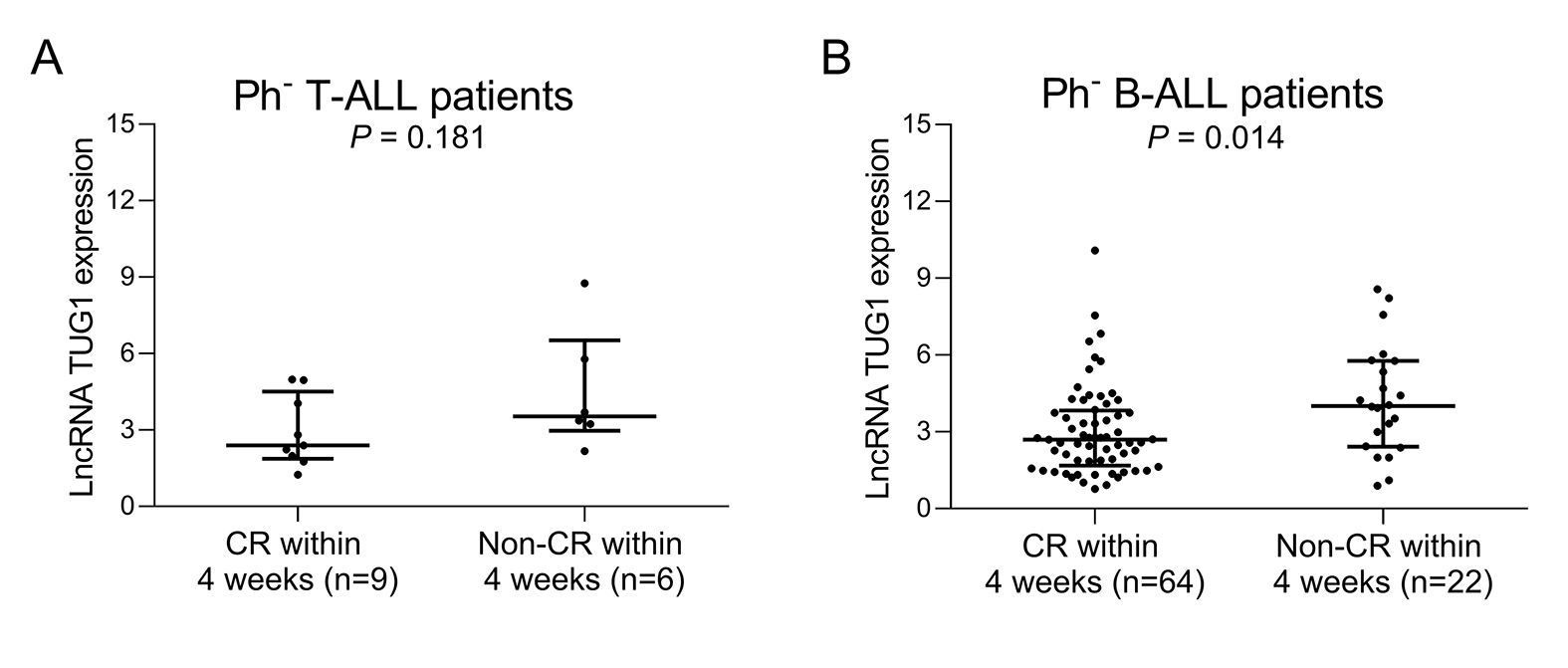

Supplement: Supplementary file 2 — Fig S2 [file JCLA-35-e23583-s002.tif]

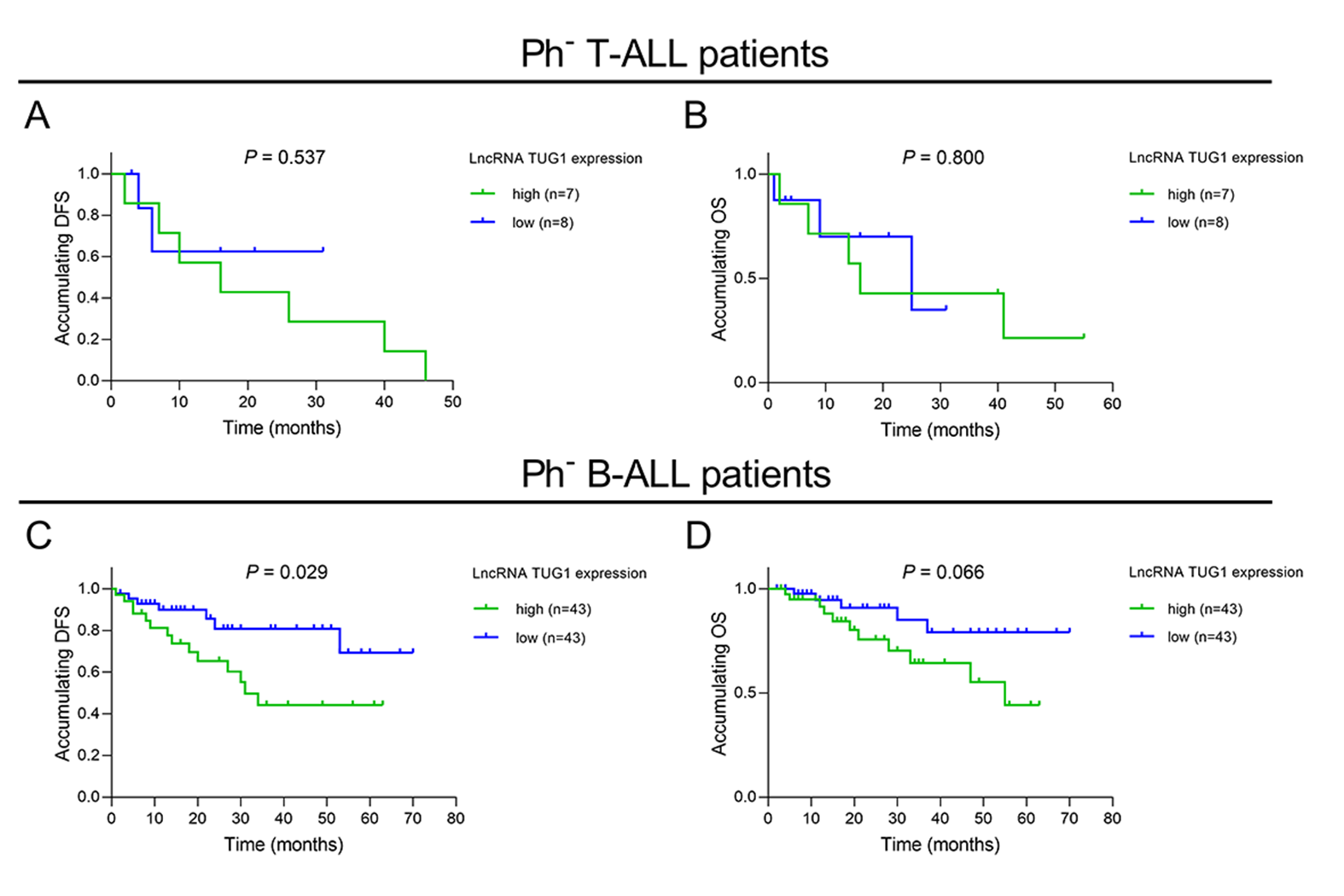

Supplement: Supplementary file 3 — Fig S3 [file JCLA-35-e23583-s001.tif]
