# Supplementary material for: Correlation analysis of long non‐coding RNA TUG1 with disease risk, clinical characteristics, treatment response, and survival profiles of adult Ph− Acute lymphoblastic leukemia
Source: J Clin Lab Anal. 2021 Jul 12;35(8):e23583. doi: 10.1002/jcla.23583 (PMC8373340; doi:10.1002/jcla.23583)
Supplement: Supplementary file 4 — Table S1 [file JCLA-35-e23583-s005.docx]

**Supplementary Table 1.** CALLG2008 protocol**^#^**

| Treatment phase | Dose | Route | Time administered |
| --- | --- | --- | --- |
| **Prophase (WBC≥50×10^9^/L)** |  |  |  |
| Prednisone | 60 mg/d | PO | D-3 to D-1 |
| Cyclophosphamide | 200 mg/(m^2^/d) | IV | D-3 to D-1 |
| **Frontline induction (4 weeks)** |  |  |  |
| VDCLP (I) regimen |  |  |  |
| Vincristine**^†^** | 2 mg/d | IV | D1, 8, 15, 22 |
| Daunorubicin**^￡^** | 40 mg/(m^2^/d) | IV | D1-3, D15, 16 |
| Cyclophosphamide | 750 mg/m^2^ | IV | D1, 15^&^ |
| L-asparaginase | 6000 IU/m^2^ | IV | D11, 14, 17, 20, 23, 26 |
| Prednisone | 1 mg/(m^2^/d) | PO | D1–14, D15–28 (2/3 dose) |
| **Early stage consolidation block** |  |  |  |
| CAM (II) regimen |  |  |  |
| Cyclophosphamide | 750 mg/m^2^ | IV | D1, 8 |
| Cytarabine | 100 mg/(m^2^/d) | IV | D1–3, D8–10 |
| Mercaptopurine/thioguanine | 60 mg/(m^2^/d) | PO | D1–7 |
| HD-MTX+L-asparaginase (III) regimen |  |  |  |
| Methotrexate | 3 g/m^2^ | IV | D1 |
| Methotrexate plus dexamethasone | 10 mg+5mg | ITH | D1 |
| L-asparaginase | 6000 IU/m^2^ | IV | D3, 4 |
| MA (IV) regimen |  |  |  |
| Mitoxantrone | 8 mg/(m^2^/d)/6 mg/(m^2^/d) | IV | D1–3 |
| Cytarabine | 750 mg/m^2^ q12h | IV | D1–3 |
| **Late stage consolidation block** |  |  |  |
| VDLP (V) regimen |  |  |  |
| Vincristine | 2 mg/d | IV | D1, 8, 15, 22 |
| Daunorubicin | 40 mg/(m^2^/d) | IV | D1-3 |
| L-asparaginase | 6000 IU/m^2^ | IV | D11, 14, 17, 20, 23, 26 |
| Dexamethasone | 8 mg/(m^2^/d) | PO/IV | D1–7, D15–21 |
| COATD (VI) regimen |  |  |  |
| Cyclophosphamide | 750 mg/m^2^ | IV | D1 |
| Vincristine | 2 mg/d | IV | D1 |
| Cytarabine | 100 mg/(m^2^/d) | IV | D1–7 |
| Teniposide | 100 mg/(m^2^/d) | IV | D1–4 |
| Dexamethasone | 6 mg/(m^2^/d) | PO/IV | D1–7 |
| HD-MTX+L-asparaginase regimen |  |  |  |
| Methotrexate | 3 g/m^2^ | IV | D1 |
| L-asparaginase | 10000 IU/m^2^ | IV | D3, 4 |
| Methotrexate plus dexamethasone | 10 mg+5mg | ITH | D1 |
| TA (VIII) regimen |  |  |  |
| Teniposide | 100 mg/(m^2^/d) | IV | D1–4 |
| Cytarabine | 100 mg/(m^2^/d) | IV | D1–7 |
| **Long-term maintenance (until 36 months from diagnosis)** | | | |
| Maintenance treatment |  |  |  |
| Methotrexate | 20 mg/(m^2^/d) | PO | Day 8 of every 1 months |
| 6-Mercaptopurine | 60 mg/(m^2^/d) | PO | Days 1–7 of every 1 months |
| Intensive treatment (MOACD) |  |  |  |
| Mitoxantrone | 8 mg/m^2^ | IV | D1, 2 of every 6 months |
| Vincristine | 2 mg/d | IV | D1 of every 6 months |
| Cyclophosphamide | 600 mg/m^2^ | IV | D1 of every 6 months |
| Cytarabine | 100 mg/(m^2^/d) | IV | D1-5 of every 6 months |
| Dexamethasone | 6 mg/(m^2^/d) | PO/IV | D1-7 of every 6 months |

**#** Central nervous system leukemia (CNSL) prevention and treatment: over 18 years old High-risk patients with age above 18 years should consider (10-12 times) craniocerebral irradiation with a total dose of 18-20 Gy; those with evidence of CNSL should be irradiated with a dose of 24 Gy, and the irradiation field was craniocerebral and spinal cord. Patients in the standard-risk group may proceed as appropriate. Patients under 18 years of age may not undergo cranial radiotherapy when CNSL was not diagnosed. CALLG2008, Chinese Acute Lymphoblastic Leukemia Cooperative Group; **†** or vindesine 4 mg/d. **￡** or idarubicin 8 mg/(m^2^/d). **&** Mesna used as rescue drug. IV, intravenous; IM, intramuscular; PO, oral; ITH, intrathecal; WBC, white blood cell.
