# Supplementary material for: Correlation analysis of long non‐coding RNA TUG1 with disease risk, clinical characteristics, treatment response, and survival profiles of adult Ph− Acute lymphoblastic leukemia
Source: J Clin Lab Anal. 2021 Jul 12;35(8):e23583. doi: 10.1002/jcla.23583 (PMC8373340; doi:10.1002/jcla.23583)
Supplement: Supplementary file 5 — Supplementary Material [file JCLA-35-e23583-s003.docx]

**Supplementary Figure 1.** Comparison of lncRNA TUG1 expression in subgroup. Comparison of lncRNA TUG1 expression between Ph^-^ T-ALL patients and healthy donors (**A**) and between Ph^-^ B-ALL patients and healthy donors (**B**). LncRNA TUG1, long non-coding RNA taurine-upregulated gene 1; Ph^-^ ALL, Philadelphia chromosome-negative acute lymphoblastic leukemia.

**Supplementary Figure 2.** Correlation of lncRNA TUG1 with treatment response in subgroup. Correlation of lncRNA TUG1 with CR within 4 weeks in Ph^-^ T-ALL patients (**A**) and . Ph^-^ B-ALL patients (**B**). LncRNA TUG1, long non-coding RNA taurine-upregulated gene 1; Ph^-^ ALL, Philadelphia chromosome-negative acute lymphoblastic leukemia; CR, complete remission; allo-HSCT, allogeneic hematopoietic stem cell transplant.

**Supplementary Figure 3.** Correlation of lncRNA TUG1 with accumulating survival in subgroup. Correlation of lncRNA TUG1 with accumulating DFS (**A**) and accumulating OS (**B**) in patients with Ph^-^ T-ALL. Correlation of lncRNA TUG1 with accumulating DFS (**C**) and accumulating OS (**D**) in patients with Ph^-^ B-ALL. LncRNA TUG1, long non-coding RNA taurine-upregulated gene 1; Ph^-^ ALL, Philadelphia chromosome-negative acute lymphoblastic leukemia; DFS, disease-free survival; OS, overall survival.
